# Mapping in an apple (*Malus* x *domestica*) F_1_ segregating population based on physical clustering of differentially expressed genes

**DOI:** 10.1186/1471-2164-15-261

**Published:** 2014-04-04

**Authors:** Philip J Jensen, Gennaro Fazio, Naomi Altman, Craig Praul, Timothy W McNellis

**Affiliations:** 1 Department of Plant Pathology & Environmental Microbiology, 211 Buckhout Laboratory, The Pennsylvania State University, 16802 University Park, PA, USA; 2 United States Department of Agriculture, Agricultural Research Service, Plant Genetics Resources Unit, 630 W. North St., 14456 Geneva, NY, USA; 3 Department of Statistics, 323 Thomas Building, The Pennsylvania State University, 16802 University Park, PA, USA; 4 Genomics Core Facility, Huck Institutes of the Life Sciences, 412 Chandlee Laboratory, The Pennsylvania State University, 16802, University Park, PA, USA

**Keywords:** Powdery mildew disease, Woolly apple aphid, Gene expression marker, DNA microarray, Genetic marker development, Quantitative PCR

## Abstract

**Background:**

Apple tree breeding is slow and difficult due to long generation times, self-incompatibility, and complex genetics. The identification of molecular markers linked to traits of interest is a way to expedite the breeding process. In the present study, we aimed to identify genes whose steady-state transcript abundance was associated with inheritance of specific traits segregating in an apple (*Malus* × *domestica*) rootstock F_1_ breeding population, including resistance to powdery mildew (*Podosphaera leucotricha*) disease and woolly apple aphid (*Eriosoma lanigerum*).

**Results:**

Transcription profiling was performed for 48 individual F_1_ apple trees from a cross of two highly heterozygous parents, using RNA isolated from healthy, actively-growing shoot tips and a custom apple DNA oligonucleotide microarray representing 26,000 unique transcripts. Genome-wide expression profiles were not clear indicators of powdery mildew or woolly apple aphid resistance phenotype. However, standard differential gene expression analysis between phenotypic groups of trees revealed relatively small sets of genes with trait-associated expression levels. For example, thirty genes were identified that were differentially expressed between trees resistant and susceptible to powdery mildew. Interestingly, the genes encoding twenty-four of these transcripts were physically clustered on chromosome 12. Similarly, seven genes were identified that were differentially expressed between trees resistant and susceptible to woolly apple aphid, and the genes encoding five of these transcripts were also clustered, this time on chromosome 17. In each case, the gene clusters were in the vicinity of previously identified major quantitative trait loci for the corresponding trait. Similar results were obtained for a series of molecular traits. Several of the differentially expressed genes were used to develop DNA polymorphism markers linked to powdery mildew disease and woolly apple aphid resistance.

**Conclusions:**

Gene expression profiling and trait-associated transcript analysis using an apple F_1_ population readily identified genes physically linked to powdery mildew disease resistance and woolly apple aphid resistance loci. This result was especially useful in apple, where extreme levels of heterozygosity make the development of reliable DNA markers quite difficult. The results suggest that this approach could prove effective in crops with complicated genetics, or for which few genomic information resources are available.

## Background

The advent of large-scale transcription profiling technologies, such as DNA microarrays [1] and RNAseq technology [2], has allowed the analysis of gene expression patterns at the genome level. DNA microarrays have been used for genetic mapping studies based on polymorphisms between parental genotypes [1]. When used to analyze genetically segregating populations, DNA microarrays have facilitated the discovery of gene expression markers [2]. Gene expression markers can be defined as transcripts encoded by genes whose relative messenger RNA expression level is inherited and segregates as a phenotypic trait [3].

Apple tree breeding is a slow and difficult process for reasons that include long juvenility periods, large size of mature plants, inbreeding depression, reproductive self-incompatibility, and complex phenotypes related to grafted trees [4]. The use of molecular markers for marker-assisted breeding and selection has the potential to expedite this process by increasing the percentage of desired genotypes and associated phenotypes early on in the breeding pipeline and assisting breeders in combining desirable traits from different parents into breeding progenies [5,6]. In the present study, our objective was to identify genes whose steady-state expression level in healthy, uninfected apple shoot tips correlated with the inheritance of agriculturally important traits in an apple rootstock breeding population. These transcripts would have the potential to be used as molecular markers by themselves, or could be used to develop DNA polymorphism markers for marker-assisted selection and gene mapping in the population.

Genetics in apple is typically done in the F_1_ generation due to the self-incompatibility of apple [7]. The apple rootstock population used for this study was an F_1_ population from a cross of two highly heterozygous rootstock parents, ‘Ottawa 3’ (O3) and ‘Robusta 5’ (R5). This is a phenotypically diverse and well-characterized population that is segregating for numerous traits of interest to apple growers. The segregating traits include resistance to biotic stresses such as fire blight (*Erwinia amylovora*; [8]) and powdery mildew (PM, *Podosphaera leucotricha*; [9]) diseases and resistance to the woolly apple aphid (WAA; *Eriosoma lanigerum*; [8,10]) pest.

We applied DNA microarray transcription profiling to 48 individual F_1_ trees from the O3 × R5 cross and identified transcripts with expression levels associated with PM disease and WAA pest resistance phenotypes. When the genes encoding these transcripts were mapped to the apple genome, they were found to be physically clustered. This is similar to the phenomenon described for single nucleotide polymorphisms and gene expression markers in *Brassica napus*[11]. The utility of using physically clustered, differentially expressed genes for DNA marker development will be discussed.

## Results

### Microarrays

**Figure 1:**
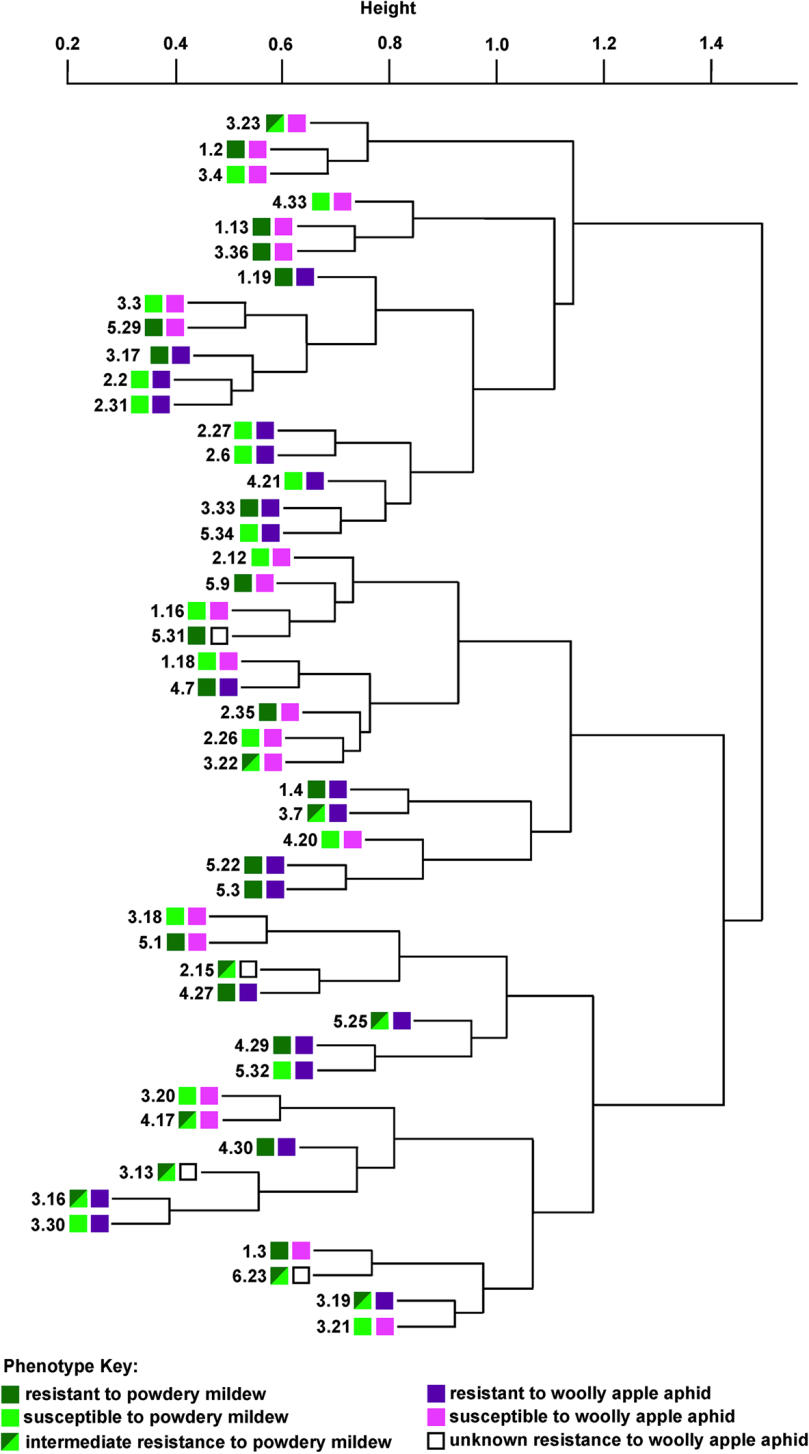
**Clustering of the 48 arrays based on their overall similarity in gene expression patterns.** The tree represented by each array is indicated by tree identification number. The powdery mildew and woolly apple aphid resistance phenotype for each tree is indicated.

RNA was isolated from healthy, uninfected, uninfested shoot tips collected from 48 individual F_1_ trees from the O3 × R5 cross population growing in a rootstock production stool bed in Geneva, NY. The RNA was used to probe 24 microarrays in multi-plex format, using two different color probes per array, so that all 48 RNA samples could be assayed on the 24 microarrays. The microarrays were clustered based on their expression profiles using the hierarchical clustering function in R (complete linkage, Figure 1). The array clustering groups did not consistently correspond with either PM resistance or WAA resistance, with closely clustered arrays often including F_1_ individuals with contrasting phenotypes for PM and WAA resistance (Figure 1).

### Physical clustering of differentially expressed genes

**Figure 2:**
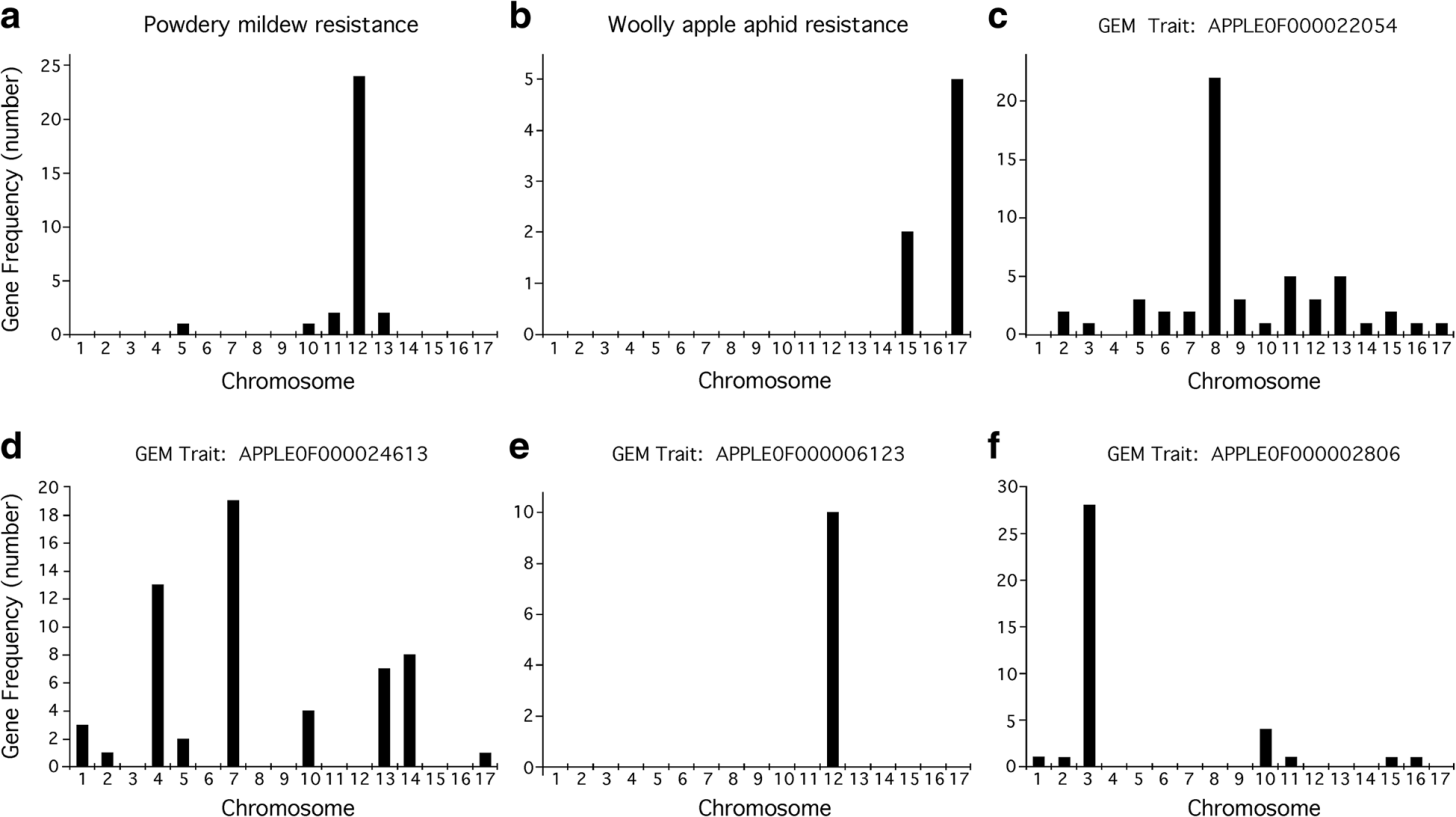
**Physical clustering of differentially expressed genes at the genome level.** Genes that were differentially expressed between phenotypic groups of trees were mapped to each of the seventeen apple chromosomes, as indicated. Phenotypic groups were developed based on resistance to powdery mildew disease **(a)**, resistance to woolly apple aphid **(b)** and several gene expression markers (GEMs, **c-f**).

**Figure 3:**
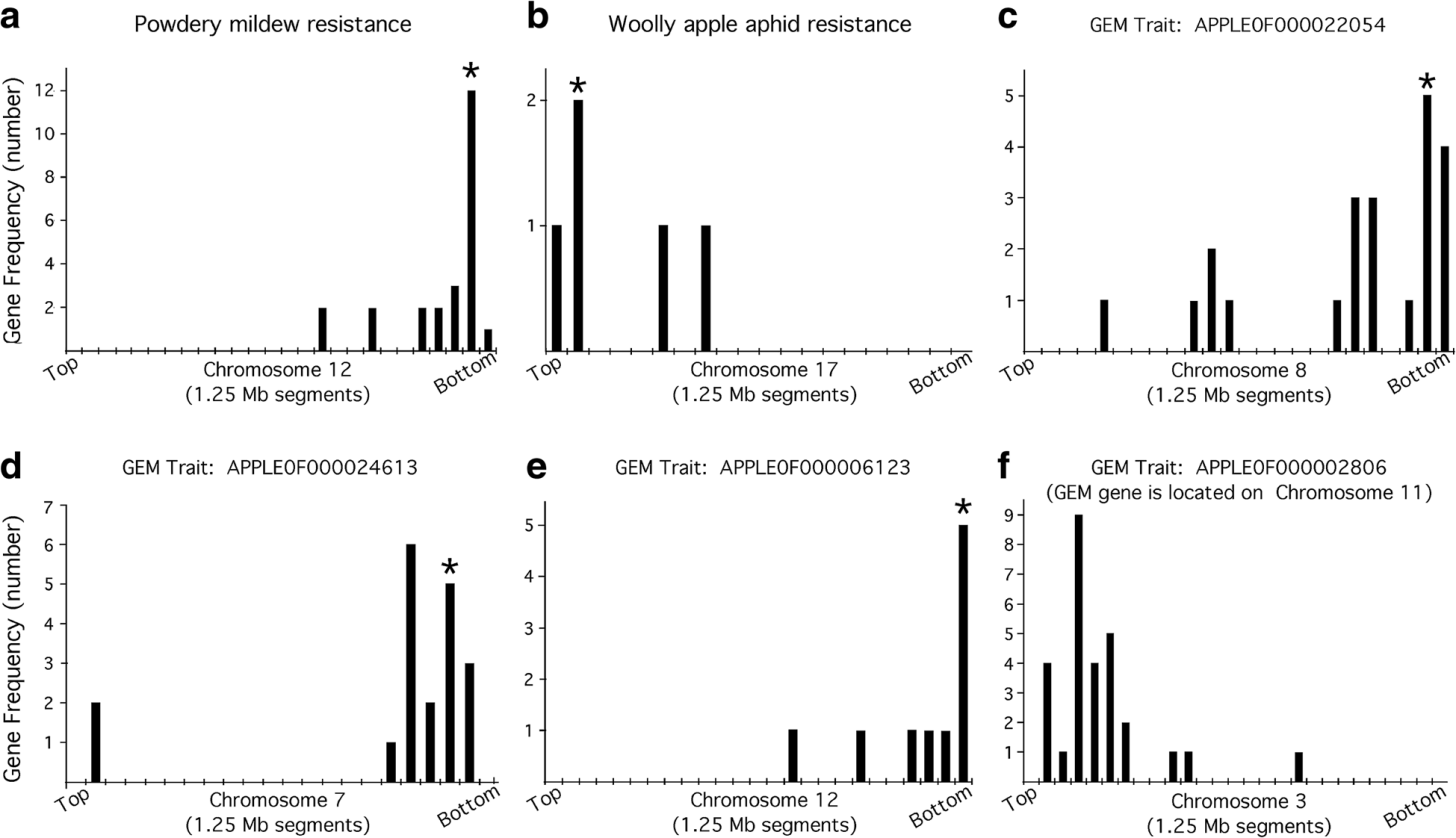
**Physical clustering of differentially expressed genes at the chromosome level.** Genes that were differentially expressed between phenotypic groups of trees were mapped on to the chromosome where they were most abundant for that trait, as indicated. The distribution along single chromosomes of genes that were differentially expressed between phenotypic groups of trees based on resistance to powdery mildew disease **(a)**, resistance to woolly apple aphid **(b)**, and four different gene expression markers (GEMs, **c-f**) are shown. Asterisk indicates chromosome segment containing the major, previously identified QTL for the trait **(a, b)** or containing the gene expression marker gene used as the molecular trait **(c-e)**.

**Figure 4:**
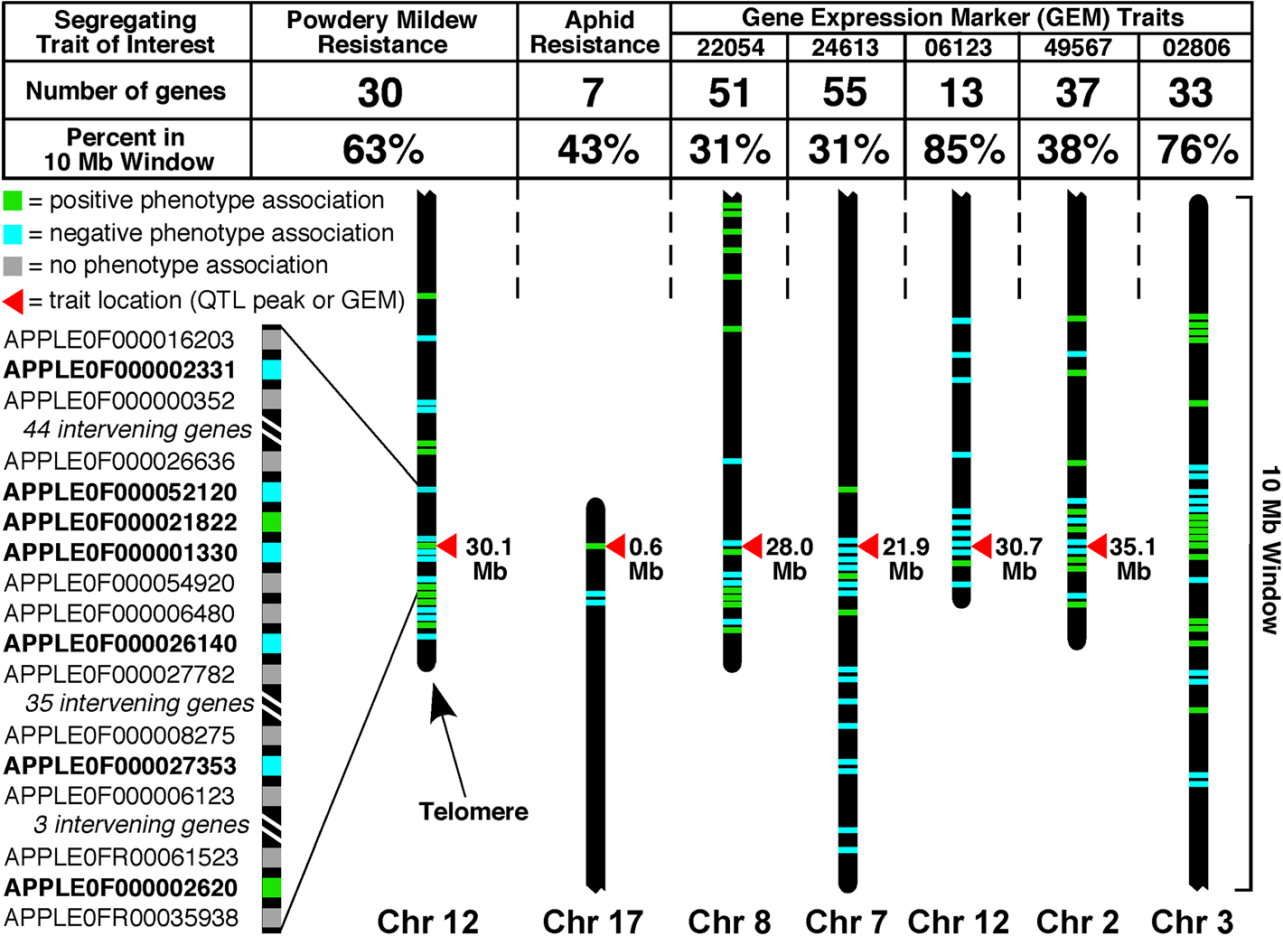
**Expression patterns of physically clustered differentially expressed genes.** For each trait, a subset of all the differentially expressed genes was located within a 10 Megabase (Mb) window centered on the physical location of the trait of interest in the apple genome. For powdery mildew resistance, a 1.6 Mb expanded window shows details, including transcript identifier numbers. Differentially expressed genes correlating with powdery mildew and woolly apple aphid resistance included some having higher expression (positive correlation) and some having lower expression (negative correlation) in resistant trees. Similarly, differentially expressed genes correlating with gene expression markers included some having higher expression (positive correlation) and some having lower expression (negative correlation) in trees where the gene expression marker gene expression level was high. Chr, chromosome.

A group of F_1_ individuals resistant to PM and a group of F_1_ individuals susceptible to PM were selected (Figure 1). Using standard differential expression analysis [12], thirty transcripts whose expression levels were differentially expressed between the PM-resistant and PM-susceptible phenotype groups were identified (q-value < 0.05; Table 1; Additional file 1: Table S1). The physical locations of the genes encoding these transcripts on the ~742 Megabase (Mb), 17-chromosome apple genome [7] were determined using the BLAST [13] server on the Genome Database for Rosaceae [14]. Twenty-four of the genes were located on chromosome 12 (Figure 2a), with nineteen being clustered within a 10 Mb region centered on the major, previously-identified PM quantitative trait locus (QTL) segregating in the in the O3 × R5 F_1_ breeding population (Figures 3a and 4).

**Table 1:** Differentially expressed genes for powdery mildew and woolly apple aphid resistance identified by q-value analysis

**Seq. ID**	Delta expression (R-S*)	**q-value**	**Malus contig hit**	**e value**	**Chromosome**	**Position (Mb)**	**Top BLAST hit annotation**	**e value**
**Powdery Mildew (PM) Resistance**								
APPLE0FR00030459	-1.47	0.028	MDC001877.277	2E-77	2	22.4	Putative far-red impaired response protein	6E-03
APPLE0F000059353	1.67	0.026	MDC012092.687	1E-173	5	15.5	Basic helix-loop-helix (bHLH) family protein contains Pfam profile: PF00010	2E-07
APPLE0FR00047019	-2.82	0.013	MDC004974.297	0.0	10	27.2	No Hits Found	
APPLE0F000058978	-1.47	0.013	MDC006524.418	1E-51	11	25.6	Disease resistance protein (TIR-NBS-LRR class)	1E-05
APPLE0F000025011	1.71	2.E-05	MDC015374.161	6E-70	11	29.2	Aldo/keto reductase family protein	3E-23
APPLE0FR00035938	1.55	0.066	MDC018304.133	0.0	13	7.1	Contig hit is MDC018304.113 at 0e-0	
APPLE0F000055730	-1.40	0.023	MDC013308.308	1E-166	13	15.9	Similar to phosphoinositide phosphatase SAC1	7E-24
APPLE0FR00044992	-1.28	0.046	MDC021221.285	1E-85	12	18.9	5′ UTR for amino acyl transferase. Contig hit is MDC021221.285 at 1e-85	
APPLE0F000015813	-1.47	0.013	MDC020309.410	7E-52	12	19.1	Short-chain dehydrogenase/reductase (SDR)	6E-56
APPLE0F000001444	-1.47	0.008	MDC010597.451	0.0	12	23.2	Potassium channel tetramerisation domain-containing	4E-05
APPLE0FR00037713	-1.76	0.017	MDC021098.161	0.0	12	23.5	Possible glycoside hydrolase	
APPLE0F000016461	1.25	0.013	MDC001938.330	0.0	12	26.6	Expressed protein	3E-08
APPLE0F000004849	-1.79	0.023	MD011583.315	0.0	12	27.1	Hydrophobic protein (RCI2B)/low temperature and salt responsive protein (LTI6B)	2E-08
APPLE0F000054055	-1.52	0.015	MDC015856.337	1E-102	12	28.1	Dormancy/auxin associated family protein similar to Auxin-repressed 12.5 kDa protein	5E-23
APPLE0F000013256	-4.76	0.066	MDC17578.59	0.0	12	28.3	Similar to histone H2A Lycopersicon esculentum	1E-33
APPLE0F000001606	3.30	2.E-12	MDC016172.153	0.0	12	28.7	Amidase family protein similar to component of chloroplast outer membrane translocon Toc64	5E-56
APPLE0FR00048809	3.59	8.E-12	MDC002462.199	6E-21	12	28.7	OSJNBa0020P07.1 [Oryza sativa] emb|CAE01284.1|	1E-01
APPLE0F000002331	-1.51	0.013	MDC016716.178	1E-131	12	29.7	Esterase/lipase/thioesterase family protein contains	1E-77
APPLE0F000052120	-1.73	1E-05	MDC011503.301	1E-90	12	30.1	Aldo/keto reductase family protein	2E-69
APPLE0F000021822	5.11	2E-09	MDC011503.301	1E-71	12	30.1	Aldo/keto reductase family protein	6E-48
APPLE0F000001330	-1.79	0.004	MDC015374.161	1E-107	12	30.1	Aldo/keto reductase family protein	7E-74
APPLE0F000026140	-1.59	0.008	MDC001112.153	1E-130	12	30.2	bZIP transcription factor family protein	4E-26
APPLE0F000027353	-1.64	0.004	MDC010137.204	1E-169	12	30.7	Pex2/Pex12 N-term. domain-containing protein similar to Peroxisome assembly protein 12	2E-05
APPLE0F000002620	1.77	2E-04	MDC012984.227	1E-152	12	30.9	Expressed protein	1E-96
APPLE0F000004618	1.64	0.001	MDC003837.156	1E-163	12	31.0	Expressed protein	3E-11
APPLE0F000004776	1.39	0.002	MDC018359.70	1E-148	12	31.1	Expressed protein	6E-46
APPLE0F000002243	-1.40	0.013	MDC018666.236	0.0	12	31.1	F-box family protein/SKP1 interacting partner 3-related	1E-33
APPLE0F000017753	-1.47	0.023	MDC001897.515	1E-137	12	31.2	Protein kinase family protein contains protein kinase domain, Pfam:PF00069	7E-09
APPLE0F000016759	2.64	1E-06	MDC014107.424	0.0	12	31.2	Ferrochelatase II identical to Swiss-Prot:O04921 ferrochelatase II,	3E-30
APPLE0F000012301	-1.58	0.004	MDC022119.74	0.0	12	31.5	Acyl carrier family protein/ACP family protein similar to SP|P53665	3E-44
**Woolly Apple Aphid (WAA) Resistance**								
APPLE0FR00041901	-0.87	0.011	MDC005468.461	2E-70	15	9.5	No Hits Found	
APPLE0FR00031359	0.96	3E-04	MDC001707.261	6E-90	15	9.5	No Hits Found	
APPLE0FR00067578	0.60	0.001	MDC0021610.17	4E-48	17	0.6	No Hits Found	
APPLE0FR00068101	-0.94	7E-04	MDC015568.269	0.0	17	1.4	No Hits Found	
APPLE00R00024612	-0.50	0.037	MDC021003.307	0.0	17	1.4	Pentatricopeptide (PPR) repeat-containing protein	8E-46
APPLE0F000027287	0.65	0.007	MDC012514.262	1E-172	17	7.2	Terpene synthase/cyclase family protein similar to myrcene/ocimene synthase	2E-15
APPLE00R00016498	-0.46	0.008	MDC008184.205	1E-179	17	9.8	Similar to ribulose-1,5-bisphosphate carboxylase/oxygenase small subunit N-methyltransferase I	3E-47

*R-S = resistant - susceptible.

Similarly, a group of F_1_ individuals resistant to WAA and a group of F_1_ individuals susceptible to WAA [8,10] were selected (Figure 1). Using standard differential expression analysis, seven transcripts that were differentially expressed between the WAA-resistant and WAA-susceptible groups were identified (Table 1; Additional file 1: Table S1). The genes encoding five of these transcripts lay on chromosome 17 (Figure 2b), all within the top 9 Mb of chromosome 17 (Figure 3b), and three of these were within about 1 Mb of a major, previously-identified WAA resistance QTL (Figures 3b and 4).

Additionally, we performed similar analyses using gene expression markers [3] with clear segregation patterns in the apple F_1_ population as molecular traits (Additional file 2: Figure S1). Trees were separated into phenotypic groups based on the expression level of the gene expression marker, with one group containing plants with high expression levels of the gene expression marker, and the other group containing plants with low expression levels of the gene expression maker. Then, genes that were differentially expressed between the two phenotypic groups were identified by standard differential expression analysis. In all cases examined, genes that were differentially expressed between phenotype groups based on gene expression markers were disproportionately located on single chromosomes (Figure 2c-f, and Additional file 1: Table S1), often clustering in the physical vicinity of the gene expression marker gene used to define the phenotypic groups (Figure 3c-e), or occasionally clustering at a separate location (Figure 3f; and Additional file 1: Table S1).

**Figure 5:**
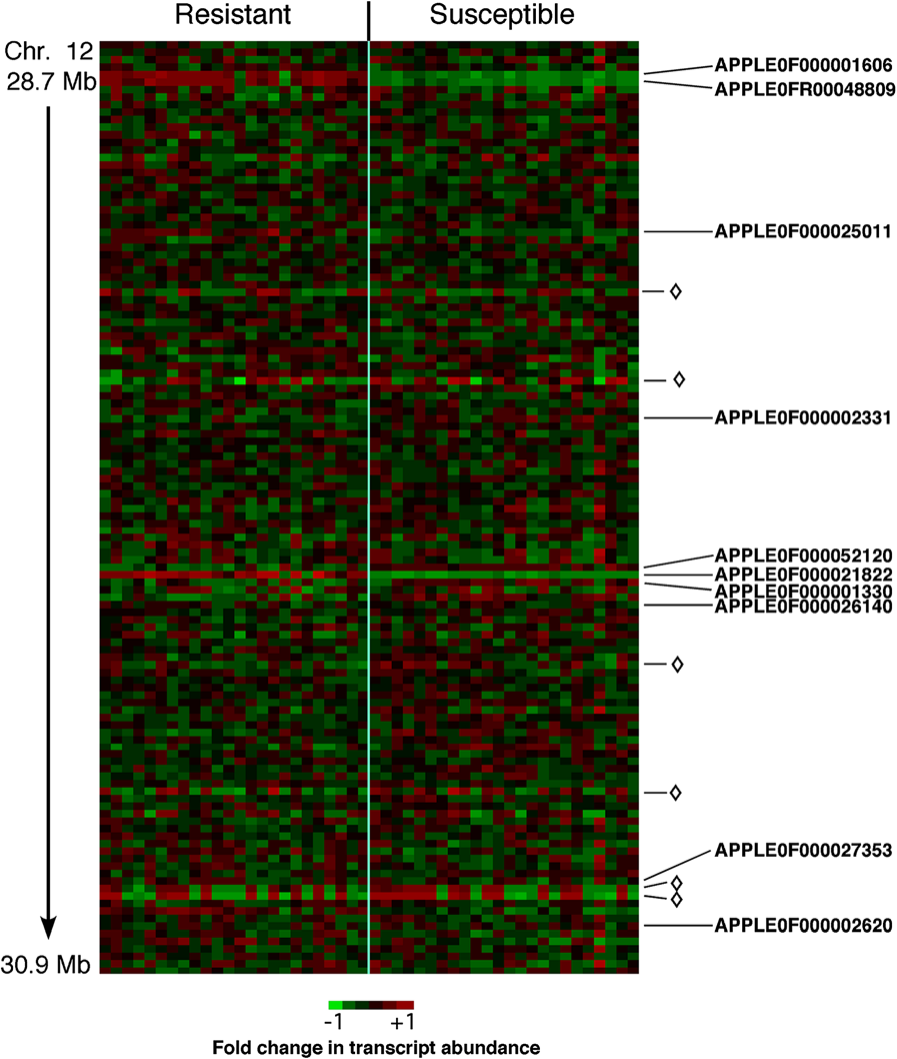
**Expression heat map of genes in the area of the powdery mildew resistance QTL.** Genes are arranged in their linear order along the chromosome, and trees are divided into groups according to powdery mildew resistance phenotype. Each column of colored blocks represents gene expression readings from one individual tree. Data for all genes queried by the microarray lying between positions 28.7-30.9 Mb of chromosome 12 are shown. Green blocks indicate trees where the expression of a given gene was lower than the average for that gene across all 48 trees; red blocks indicate plants where the expression of a given gene was higher than the average for that gene across all 48 trees. Genes differentially expressed between the powdery mildew disease resistance phenotype groups are indicated by sequence ID numbers. Gene expression markers with segregation expression patterns that did not correlate with powdery mildew disease resistance are denoted by diamonds. Chr, chromosome.

The expression levels of the physically clustered, differentially expressed genes had a mixture of positive and negative associations with their associated trait (Figures 4 & 5). For example, some of the physically clustered genes with expression levels associated with PM resistance had higher expression levels in the resistant trees (positive phenotype association), while others had lower expression levels in the resistant trees (negative phenotype association), and the expression levels of intervening genes had no phenotype association (Figures 4 & 5). In some instances, differentially expressed genes with positive and negative phenotype associations were in juxtaposition (Figures 4 & 5) with as little as 90 kb distance between them (Additional file 1: Table S1). Gene expression marker genes with expression patterns not associated with the PM resistance phenotype were visible within the PM QTL region (Figure 5).

It is notable that the genes with trait-associated expression levels did not necessarily have the same expression pattern in all individuals in a phenotype group. For example, PM resistance-associated genes APPLE0F000001606, APPLE0FR00048809, and APPLE0F000052120 are visibly quite consistent in their expression within a phenotypic group, while APPLE0F000026140 and APPLE0F000002331 are less consistent within each phenotypic group (Figure 5).

### Validation of gene expression level heritability and consistency

The heritability of expression level of selected genes with trait-associated expression levels was validated by quantitative polymerase chain reaction (qPCR) analysis using the O3 and R5 parents and a group of F_1_ individuals in the O3 × R5 F_1_ population growing in a location different from the trees used for DNA microarray analysis and sampled during a different year. qPCR analysis showed that PM resistance-associated gene APPLE0FR00048809 had higher expression in parent R5 compared to parent O3 (Additional file 2: Figure S2), just as predicted by the microarray experiment. Furthermore, expression of APPLE0FR00048809 among the O3 × R5 F_1_ population used for qPCR analysis segregated at a 1:1 ratio (Additional file 2: Figure S2), just as predicted by the DNA microarray data. In addition, a gene expression marker that had a distinctly bimodal expression in the DNA microarray analysis (APPLE0F000001974) also had bimodal expression in the O3 × R5 F_1_ population used for qPCR validation; this bimodal expression segregation was visible among PCR amplicons (Additional file 2: Figure S3). Finally, the relative gene expression level relationships between APPLE0FR00068101 and several genes with associated expression patterns and which exhibited continuous expression level distribution in the array data (APPLE00R00024612, APPLE0F000011491, APPLE0F000050102) were maintained in the qPCR relative expression data (Additional file 1: Tables S2 and S3).

### Development of DNA markers based on genes with trait-associated expression level

As a proof of concept, DNA markers were developed for WAA resistance-associated gene APPLE0FR00068101. The sequence of APPLE0FR00068101 was used to identify *Malus* × *domestica* contig MDC015568.236 (6,831 bp long), which contained the best matching DNA sequence according to BLAST analysis. Several polymorphisms were identified within this region when comparing the O3 haplo-contigs with the R5 haplo-contigs. Of particular interest for easy marker development were two microsatellite regions between bases 2,500 - 3,500 of contig MDC015568.236, for which PCR primer pairs were designed (Waa68101-236ssr, forward primer 5′-GGGTTGAAGTGCGAGAC-3′, reverse primer 5′-CACGCGACGAGGTATTCCAAC-3′; and Waa68101-236Indel, forward primer 5′-CCAAATTATGCATACAGATG-3′, reverse primer 5′-GATTAATGATTAGAAGAAC-3′) and tested with parent DNA with annealing temperature gradient PCR (Additional file 2: Figure S4). Both markers were polymorphic between the parents, but only the Waa68101-236ssr was heterozygous in the R5 parent, showing bands at approximately 360 bp, 460 bp and 520 bp (Additional file 2: Figure S4). Segregation analysis in the O3 × R5 population showed very strong association of the Waa68101-236ssr 360 bp band with resistance (p = 0.0001). The Waa68101-236ssr SSR marker was more predictive of WAA resistance in the O3 × R5 population than the published interval containing the R5-derived *Er2* gene delineated between SSR markers GD96 (MDC021359.285 at 11,796 Kb on Chr17) and GD153 (MDC013709.214 at 9,138 Kb on Chr17) [7,10].

## Discussion

The results of array clustering for the 48 microarrays indicated that overall gene expression patterns of individual plants were not robust indicators of PM or WAA resistance phenotype. In contrast, the differential gene expression analysis based on phenotypic groups of F_1_ individuals yielded relatively small numbers of genes that were differentially expressed between the phenotypic groups, and these differentially expressed genes displayed a remarkable degree of physical clustering on the apple genome. The clustered genes were typically in the physical vicinity of the major locus controlling the trait, in the case of PM and WAA resistance, or in the vicinity of the gene expression marker used as the molecular trait. Clusters at locations unlinked to their corresponding trait of interest (Figure 3f; and Additional file 1: Table S1) might represent the locations of other QTLs related to the phenotype. All of the phenotypes examined in this study were controlled by single, major, dominant QTL, which allowed detection of linkage using only 48 F_1_ individuals. Analysis of larger numbers of individuals would certainly be required in order to analyze multi-locus traits effectively.

The clustered differentially expressed genes are not necessarily involved in controlling their associated phenotype. For example, the differentially expressed genes associated with PM resistance did not show any obvious functional patterns or similarities (Table 1). It is also important to note here that differentially expressed genes we examined here were not selected based on their induced expression during pathogen or insect interaction. Rather, the differentially expressed genes represented transcripts whose steady-state expression levels in healthy tissue were associated with PM or WAA resistance phenotype status. It is possible that examining differential gene expression using infected or infested samples might mask the clustering due to the large numbers of genes being up- and down-regulated in response to the stress.

The clustering pattern of differentially expressed genes is consistent with the relative expression levels of these genes being inherited from a parent. This is different from genome neighborhood effects, where groups of linked genes are typically up- or down-regulated together [15]. Just as DNA polymorphism markers can be linked to a trait locus, expression patterns of some of the nearby genes are also linked. By grouping trees according to an inherited trait of interest, one might expect that the differentially expressed genes would be identified simply due to their decreased expression variation within a particular pool compared to non-correlating genes at loci unlinked to the trait of interest. However, it is remarkable that the differential expression patterns between the phenotypic groups included so few genes, and that so many of these were physically clustered. This suggests that heritable differences in gene relative expression were predominantly detected by the analysis, rather than genes whose expression levels might contribute to or be necessary for the development of the phenotype. Such genes would be expected to be scattered randomly across the genome. Our results are similar to those seen in *Brassica napus* using single nucleotide polymorphisms (SNPs) and gene expression markers [11]. However, the present study used a segregating apple tree F_1_ population, while the other study used a collection of accessions.

Validation of the expression patterns using qPCR indicated that most of the genes indeed had patterns of expression consistent with the array data. The congruence of DNA microarray and qPCR data for selected differentially expressed genes and gene expression markers provided strong validation for the DNA microarray data. qPCR validation was successful using a different set of individuals from the same cross in a different environment and year from those used for the microarray, indicating that the differentially expressed genes had relative expression levels consistent across different growing conditions and years and between different groups of individuals from the O3 × R5 F_1_ population. The development of PCR based molecular markers associated with several of the differentially expressed sequences was in many cases successful because sequence mutations such as large INDELs and microsatellite variation was discovered within or nearby the target genes. While several methods are available to detect polymorphisms in marker assisted breeding, markers based on the polymerase chain reaction are still the most accessible and least expensive for small scale breeding programs. The combination of expression analysis for target identification and sequence based marker development proved a good strategy as the PCR markers developed in this study have been routinely proven useful in apple rootstock breeding program in Geneva, NY. As RNAseq methods become more refined, it may be possible to find differentially expressed genes associated with traits of interest and at the same time leverage polymorphisms contained in the expressed sequences for haplotype specific breeding marker development.

## Conclusion

We have shown in a segregating population from the cross of a highly heterozygous plant that gene expression analysis can result in identification of differentially expressed genes that are physically linked to one another. Gene expression heritability as a method to detect genes physically linked to trait loci of interest could be useful in crops for which few genetic and genomic information resources are available. Even in the absence of a genetic map, molecular markers linked to traits of interest could be developed using transcriptome profiles of segregating populations, since a substantial proportion of the differentially expressed genes would be expected to cluster in the vicinity of the trait of interest. While there may be no causal link between the differentially expressed genes and their associated traits, they do provide an excellent starting point for development of DNA markers linked to segregating traits of interest.

## Methods

### Plant materials

The trees used for the DNA microarray analysis were from a segregating F_1_ population from an O3 × R5 cross and were grown in an orchard in Geneva, NY [16]. Samples were taken in early summer of 2009 from healthy, uninfected, uninfested individual shoots from first-year growth of 48 plants in a propagation stool bed. Shoot tips samples comprised all shoot tissues up to and including the first fully-expanded leaf. Sampled shoots were carefully selected so that they were as similar to each other as possible in size and shape to minimize sampling variation. The samples were flash frozen in liquid nitrogen and stored at -80°C for later RNA isolation.

For the qPCR analyses, shoot tip samples were collected in late spring of 2013 from a separate group of 46 individuals belonging to the same O3 × R5 population from clonally propagated material in a replicate orchard in Geneva, NY. Shoot tips from the population parents (O3 and R5) were collected from trees at the apple collection of the USDA ARS Plant Genetic Resources Unit in Geneva, NY.

### RNA isolation and microarray analysis

Total RNA was isolated from whole apple shoot tips as previously described [17]. The microarray data used in the present study were generated during a previously-reported study [18] and subjected to a new analysis. The contig sequences used for array probe development are accessible at the Gene Expression Omnibus (GEO) dataset website [19]. The array used was a second-generation array in a 12-plex array format containing 135,000 probes per plex, representing 26,017 transcripts, enabling us to query a relatively large number of samples. Each transcript was queried by 4–5 probes of 60–70 bases in length. The array included the best-performing probes from the first-generation array and was enriched for differentially-expressed genes based on analyses of the first-generation array [17]. The genes predicted to encode the 26,017 transcripts probed by the array represented were evenly and randomly physically distributed across the apple tree genome. The expression levels for each individual F_1_ tree were analyzed using a single array only; however, analyses were conducted using pooled data from trees with similar phenotypes, which represented pseudo-replicates in this context [20].

While the parents of the breeding population were different from the varieties used to design the DNA microarray, this did not interfere with probe performance or account for patterns. Any nucleotide polymorphisms between the probes and the samples, when present, did not affect hybridization, as the other probes for the same transcripts, which had no polymorphisms, gave similar intensity values (Additional file 1: Table S4). In addition, mismatches between probes and samples did not correlate with variation in between probe signals (Additional file 1: Table S4). For example, the probes for APPLE0FR00039157 had 1 or 5 mismatches to their target per probe, yet they produced data with similar signal intensity and standard deviations to the APPLE0FR00031686 probe set, which had no mismatches to their target (Additional file 1: Table S4).

### Differential gene expression analysis

The gene expression data from the DNA microarray hybridization experiments were previously normalized using R software [18]. To identify differentially expressed genes based on PM resistance phenotype, for example, the trees were divided into two groups, one group containing the PM-resistant trees, and the other group containing the PM-susceptible trees. The mean of the log_2_ (expression) value for each transcript was then calculated separately in each phenotypic group, and then the M-value (log_2_ fold difference in expression) for each transcript was computed as the difference in the mean log_2_ (expression) value for each transcript between the two groups of trees. Empirical Bayes ANOVA analysis [21] was performed using the LIMMA (Linear Models for Microarray Data) package [22] as part of the R Bioconductor suite [23]. P-values (false positive rate) from this analysis were then converted to q-values (false discovery rate) using the methodology of Storey and Tibshirani [11] as implemented in the Bioconductor q-value routine [24]. Differentially expressed transcripts were identified as transcripts with statistically different levels of expression between the phenotypic groups (q-value < 0.05), regardless of the magnitude of the difference (M-value). Differentially expressed genes for WAA and the several GEM traits were identified using the same approach, using phenotypic groups defined by each trait.

### Identification of gene expression markers

The first step was to calculate the mean of the log_2_ (expression) value for each transcript in the entire F_1_ population. Next, the divergence in expression for each individual tree compared to the average was determined for each transcript. GEMs were identified as transcripts that could divide the trees into groups of roughly equal size based on having at least a 1.5-fold difference in expression levels between the groups (Additional file 2: Figure S1).

### Expression pattern validation by qPCR

Shoot tip samples were processed fresh, immediately after collection, using the ZR Plant RNA MiniPrep kit (Zymo Research, Irvine CA, USA) following the manufacturer’s instructions, with the addition of DNAse I (Invitrogen, Grand Island, NY, USA) to the RNA wash buffer in the kit as recommended in the manufacturer’s protocol. cDNA synthesis was performed using the QuantiTect Reverse Transcription kit (Qiagen, Germantown, MD, USA) according to the manufacturer’s instructions. PCR primers for selected differentially expressed genes and gene expression markers were optimized using parental genomic DNA with Annealing Temperature Gradient PCR (ATG-PCR; Additional file 1: Table S5). qPCR was performed in 25 μl reactions using LightCycler 480 SYBR Green I Master reaction mix (Roche, Indianapolis, IN, USA) and a LightCycler 480 instrument (Roche) A Basic Relative Quantification workflow was used that included fluorescence measurement at each PCR extension step and a final melting step measuring fluorescence from 95-55°C for Melt Curve Genotyping. An actin gene (Genbank accession number EB 136338, primers: 5′-GGCTGGATTTGCTGGTGATG-3′ and 5′-TGCTCACTATGCCGTGCTCA-3′) was used as the reference for Relative Quantification Analysis (RQA). The Crossing Point (Cp) values and ratios between target and references were calculated using the LC480 software and algorithms (Roche). Melt curves were analyzed for non-specific amplification peaks (Additional file 2: Figure S2). Amplicons were resolved on 1.5% ethidium bromide (EtBr) stained agarose gels and visualized with an AlphaImager HP gel documentation system (ProteinSimple, Santa Clara, CA, USA).

### DNA marker development

The sequence of gene APPLE0FR00068101, whose expression was associated with WAA resistance, was compared to the ‘Golden Delicious’ apple genome hosted by the Genome Database for Rosaceae [14] using the National Center for Biotechnology Information (NCBI)’s BLASTN program [12] to identify four contigs (MDC015568.236, MDC013761.427, MDC015568.269, MDC000748.724) containing similar sequences (e-values between 1E-97 to 2E-89). All four contigs were located on apple chromosome 17 within a 74 kb interval (genome base pair positions 1,405,743-1,479,871; Additional file 2: Figure S5). Genomic sequences for parents O3 and R5 had been obtained by Next-Gen Illumina Hi-Seq paired end sequencing. Geneious bioinformatic software (Biomatters, San Francisco, CA, USA) was used to construct a local alignment of next-gen sequences to the *Malus* × *domestica* contig containing the complete predicted target gene sequence (MDP). Contig MDC015568.236 contained sequences most similar to the R5 next-gen sequences, and was therefore chosen for further analysis. Unique single nucleotide polymorphisms (SNPs), simple sequence repeats (SSRs), and haplotypes were identified for the R5 (WAA-resistant) parent. Several 18–21 bp PCR primers were designed to match unique SNP haplotypes at the 3′ end, and primer pairs were tested with parental DNAs using annealing temperature gradient PCR (45°C to 65°C) to verify genotype specificity, stability and reproducibility of amplicons. Amplicons were resolved on 2% EtBr-stained agarose gels and visualized with an AlphaImager HP gel documentation system (ProteinSimple). In addition, some primers were designed flanking microsatellite SSRs. Genotype-specific amplicons were then tested on segregating individuals in the O3 × R5 population and a diversity panel of apple rootstocks to verify genetic inheritance, linkage to other markers, and haplotype uniqueness.

### Availability of supporting data

Microarray data are available through the GEO website using accession number GSE43268.

## Abbreviations

ANOVA: Analysis of variance; ATG-PCR: Annealing temperature gradient PCR; BLAST: Basic local alignment search tool; bp: Base pairs of DNA; cDNA: Complementary DNA; DNA: Deoxyribonucleic acid; EtBr: Ethidium bromide; kb: Kilobase pairs; O3: ‘Ottawa 3’; PCR: Polymerase chain reaction; PM: Powdery mildew; qPCR: Quantitative PCR; QTL: Quantitative trait locus; R5: ‘Robusta 5’; RNA: Ribonucleic acid; RNAseq: RNA sequencing; SNP: Single nucleotide polymorphism; SSR: Simple sequence repeats; USDA: United States Department of Agriculture; WAA: Woolly apple aphid.

## Competing interests

The authors declare that they have no competing interests.

## Authors’ contributions

PJJ performed the microarray experiments and analyzed the results; GF performed phenotypic characterizations of apple trees and performed the qPCR validation of selected transcripts and developed the DNA markers based on the differentially expressed genes; NA provided statistical advice and assisted with data analysis; CP performed microarray hybridizations and provided advice for experiment design and data interpretation; TWM initiated, developed, and supervised the project; all authors contributed to the writing of the manuscript and have approved the manuscript.

## Supplementary Material

Additional file 1: Table S1 Transcripts identified as having expression patterns associated with powdery mildew resistance, woolly apple aphid resistance, and the gene expression marker traits analyzed in a segregating F_1_ population from an ‘Ottawa3’ × ‘Robusta 5’ cross. **Table S2.** Correlation between Microarray Gene Expression Values for WAA resistance differentially expressed genes APPLE0FR00068101, and associated features APPLE00R00024612, APPLE0F000011491, APPLE0F000050102. **Table S3.** Correlation between Relative Gene Expression values (Target Gene/Actin Reference Gene) in qPCR Basic Relative Quantification outputs for the following targets: APPLE24612, APPLE50102, APPLE11491 and WAA resistance differentially expressed gene APPLE68101. **Table S4.** Individual probe data and SNP counts for transcripts in the region 29.0 to 30.2 Mb on Chromosome 12. **Table S5.** qPCR conditions and primers for gene expression validation. Click here for file

Additional file 2: Figure S1 Example gene expression marker (GEM) trait. **Figure S2.** Relative quantification results for qPCR of differentially expressed gene APPLE0FR00048809 (associated with PM resistance) relative to actin. **Figure S3.** Visualization of qPCR amplicons of gene APPLE0F000001977, showing clear segregation (presence/absence) of amplified target cDNA in selected progeny. **Figure S4.** Annealing temperature gradient amplification (65°C - 45°C) of differentially expressed gene APPLE0FR00068101 derived markers on parental DNAs. **Figure S5.** Alignment of microarray feature APPLE0FR00068101 to Chromosome 17 of the apple genome. Click here for file
