# Supplementary material for: Mapping in an apple (Malus x domestica) F1 segregating population based on physical clustering of differentially expressed genes
Source: BMC Genomics. 2014 Apr 4;15:261. doi: 10.1186/1471-2164-15-261 (PMC4051173; doi:10.1186/1471-2164-15-261)
Supplement: Additional file 2: Figure S1 — Example gene expression marker (GEM) trait. Figure S2. Relative quantification results for qPCR of differentially expressed gene APPLE0FR00048809 (associated with PM resistance) relative to actin. Figure S3. Visualization of qPCR amplicons of gene APPLE0F000001977, showing clear segregation (presence/absence) of amplified target cDNA in selected progeny. Figure S4. Annealing temperature gradient amplification (65°C - 45°C) of differentially expressed gene APPLE0FR00068101 derived markers on parental DNAs. Figure S5. Alignment of microarray feature APPLE0FR00068101 to Chromosome 17 of the apple genome. [file 1471-2164-15-261-S2.PDF]

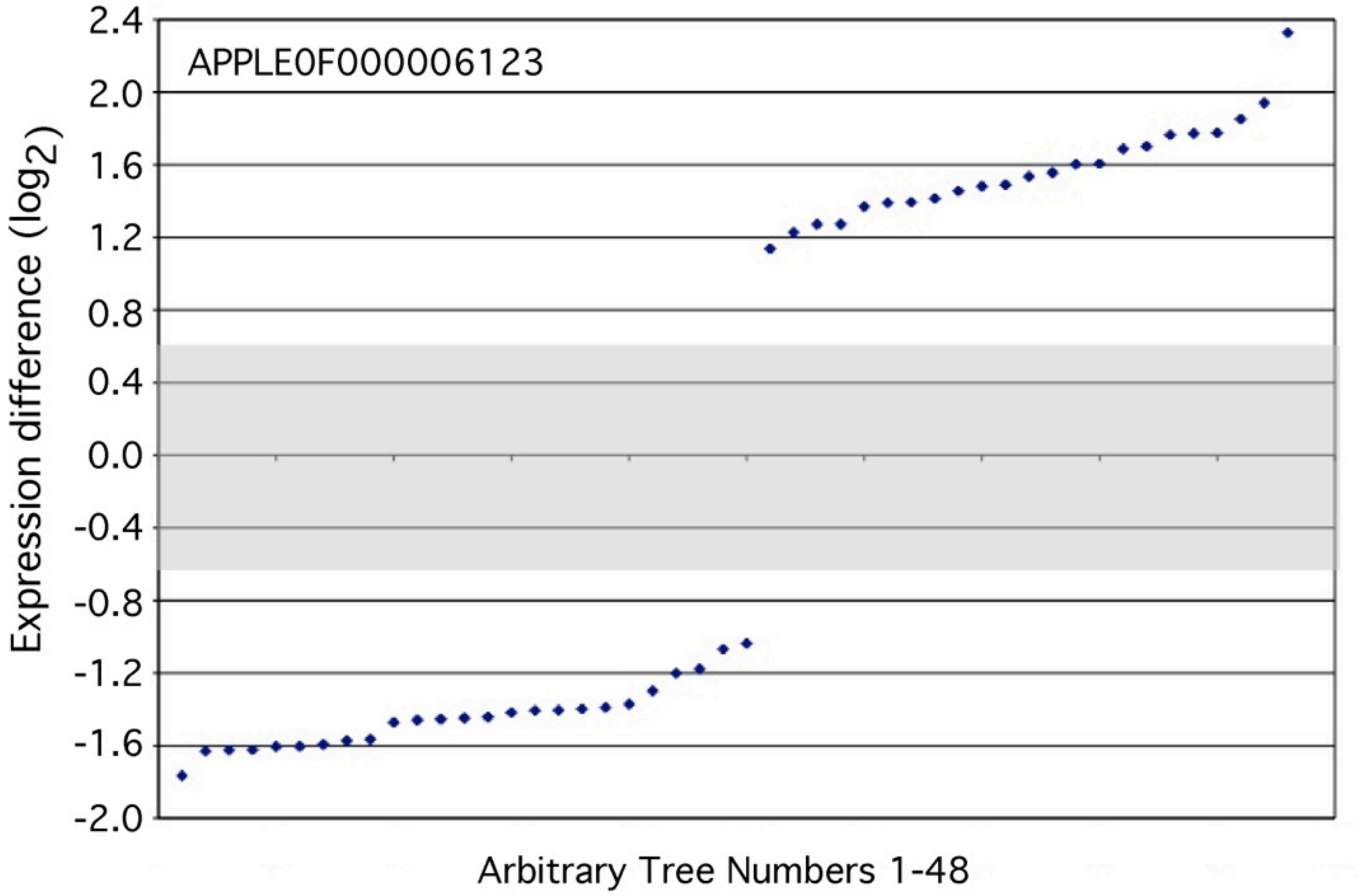

Figure S1
Jensen et al.
BMC Genomics

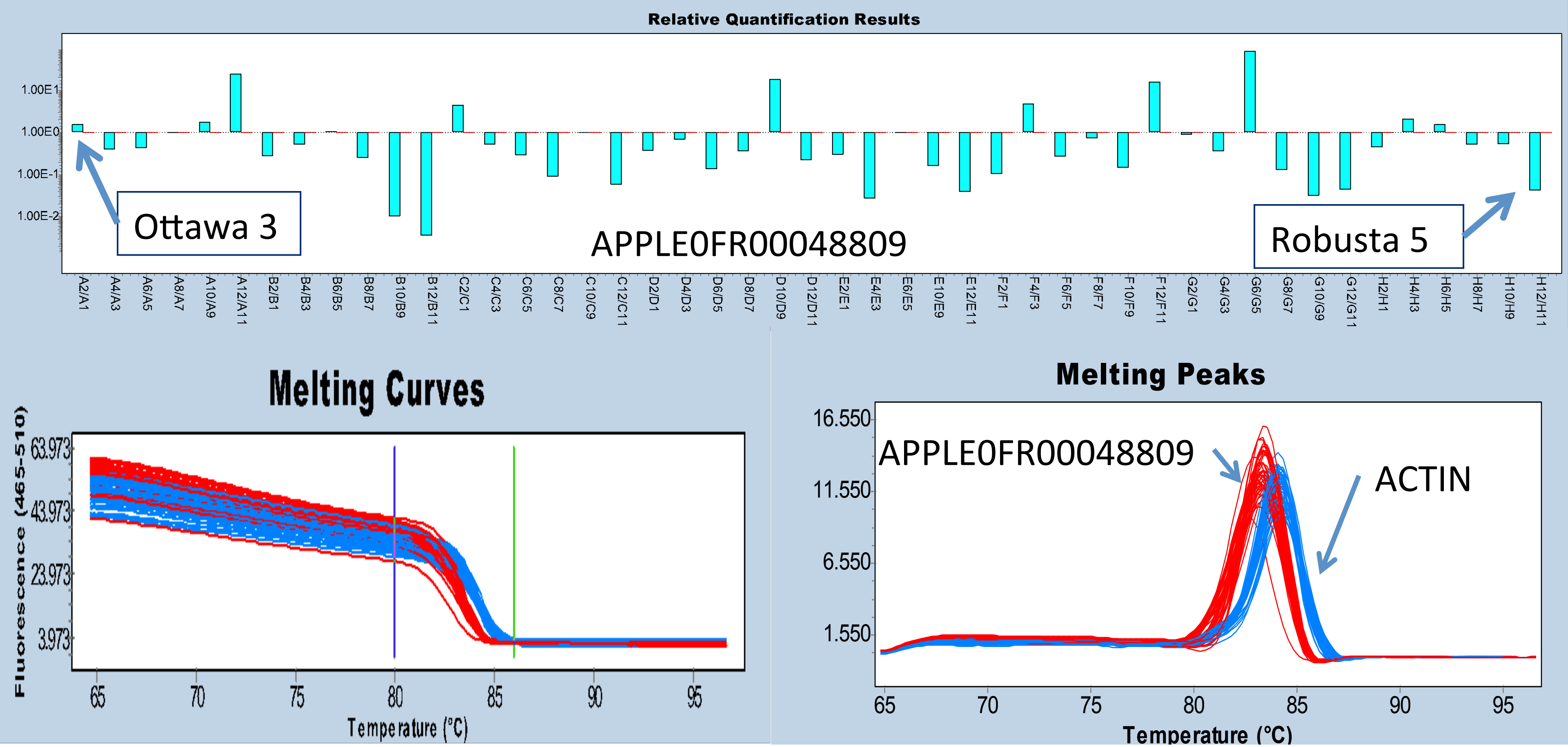

Figure S2.
Jensen et al.
BMC Genomics

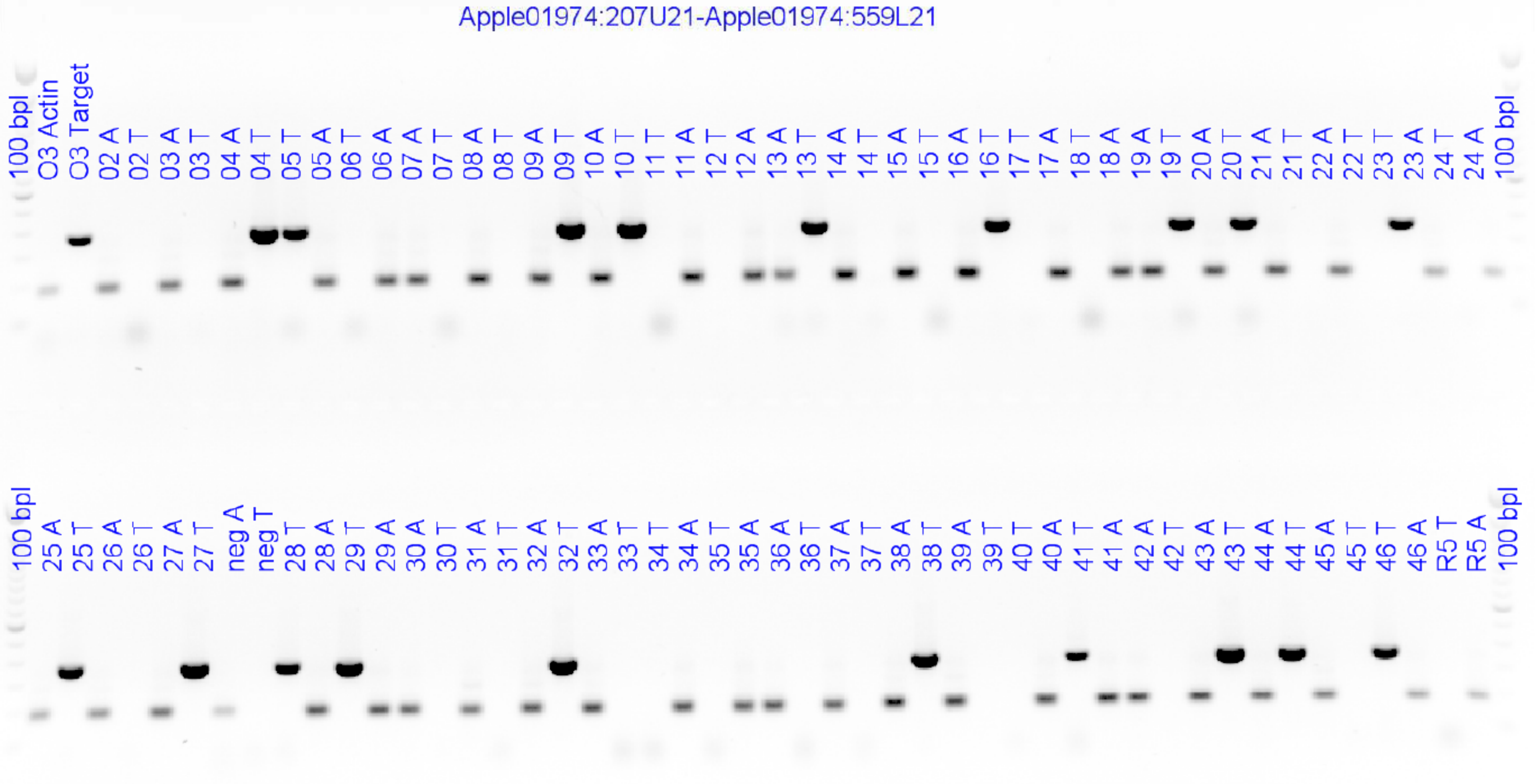

Figure S3.
Jensen et al.
BMC Genomics

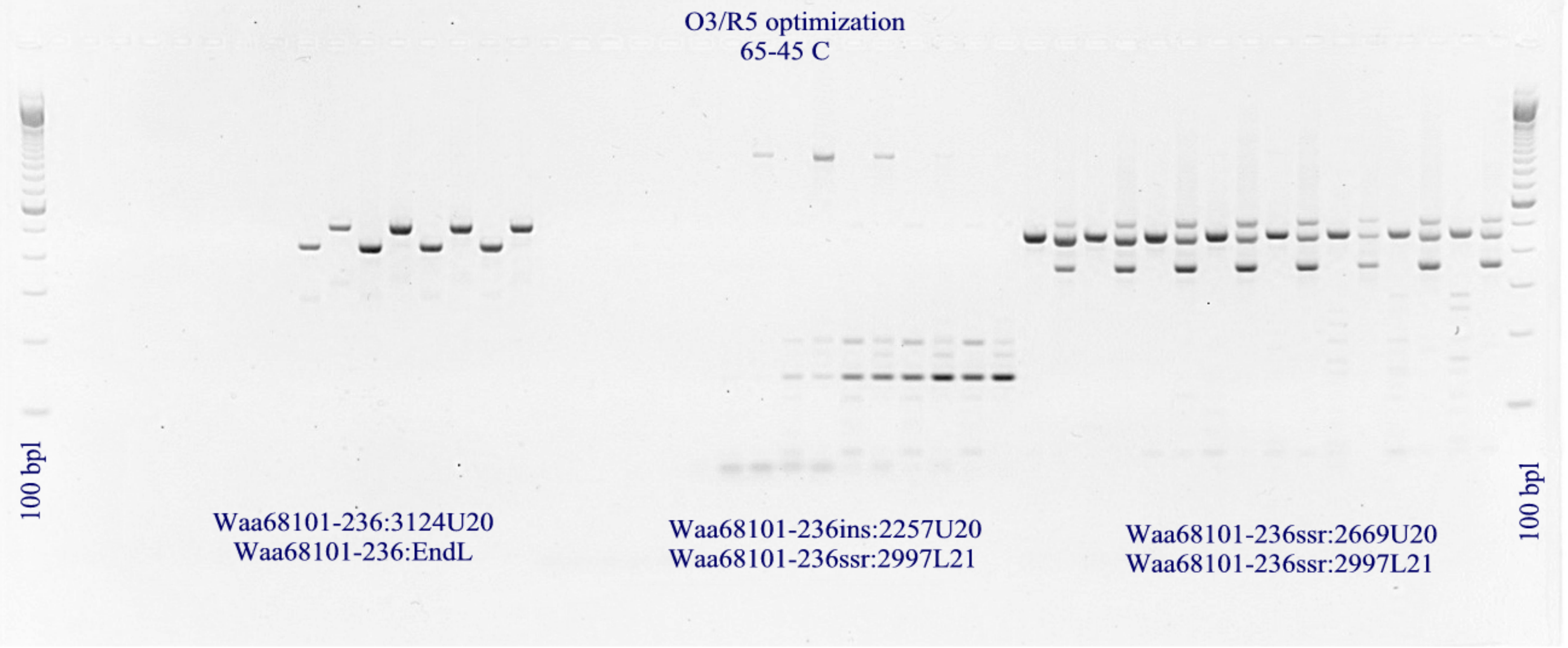

Figure S4.
Jensen et al.
BMC Genomics

Figure S5 Alignment of microarray feature APPLE0FR00068101 to Chromosome 17 of the apple genome displayed in Genome Browser at the Genome Database for Rosaceae (www.rosaceae.org) and the BLAST alignments to the four contigs (MDC015568.269, MDC000748.724, MDC015568.236, MDC013761.427) that matched the best with it.

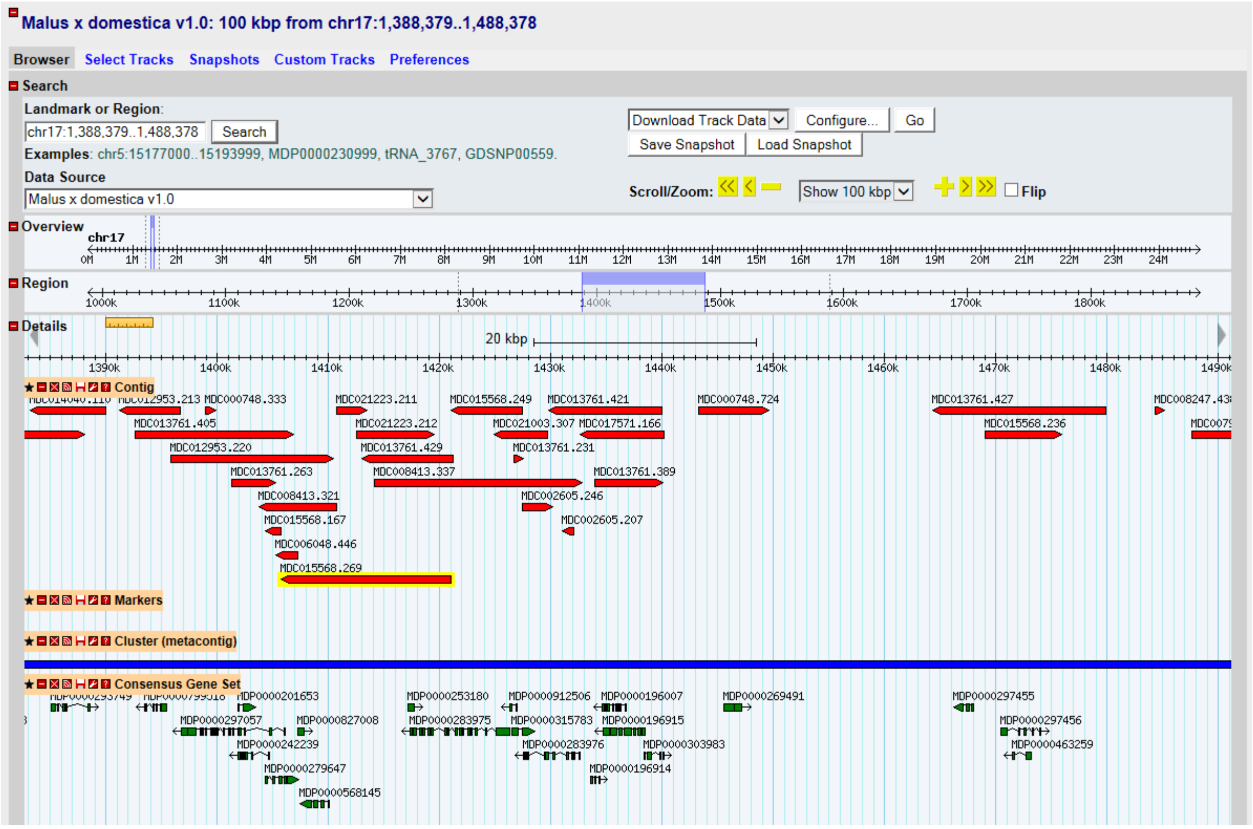


```
Sequences producing significant alignments:                    (bits)  Value

MDC015568.269 chr17:1405743..1421014                             682    0.0
MDC000748.724 chr17:1443321..1449646                             682    0.0
MDC015568.236 chr17:1469063..1475893                             379    e-103
MDC013761.427 chr17:1464328..1479871                             379    e-103


>MDC015568.269 chr17:1405743..1421014
          Length = 15272

 Score =  682 bits (344), Expect = 0.0
 Identities = 353/356 (99%)
 Strand = Plus / Minus


Query: 1    aaccaaactgtagcacacaattcatcactgctatgacagctaagacattccaaatcacac 60
            ||||||||||||||||||||||||||||||||||||||||||||||||||||||||||||
Sbjct: 3311 aaccaaactgtagcacacaattcatcactgctatgacagctaagacattccaaatcacac 3252
```

```
Query: 61   aagtttacatacattgggataaaacccacagttattcattgttaaatataattacaataa 120
            ||||||||||||||||||||||||||||||||||||||||||||||||||||||||||||
Sbjct: 3251 aagtttacatacattgggataaaacccacagttattcattgttaaatataattacaataa 3192


Query: 121  gttgattttgttggatgcaaccatccaactactatgatccttcagcgcccaatgttgga 180
            ||||||||||||||||||||||||||||||||||||||||||||||||||||||||||||
Sbjct: 3191 gttgattttgttggatgcaaccatccaactactatgatccttcagcgcccaatgttgga 3132


Query: 181  ttttcaaaggcgaagaaactccatactcaaaatcgtgggcatcggctggagagagattgt 240
            ||||| ||||||||||||||||||||||||||||||||||||||||||||||||||||||
Sbjct: 3131 ttttctaaggcgaagaaactccatactcaaaatcgtgggcatcggctggagagagattgt 3072


Query: 241  cttcgcgtcttgggttgaagtgcgagaccttgaagaatatgagaactggatcatctgggt 300
            ||||||||||||||||||||||||||||||||||||||||||||||||||||||||||||
Sbjct: 3071 cttcgcgtcttgggttgaagtgcgagaccttgaagaatatgagaactggatcatctgggt 3012


Query: 301  cctcttcttcttattattctttcttcaaaagtttgtaactcttatggttagagaga 356
            ||||||||||||||||||||||||||||| | ||||||||||||||||||||||||
Sbjct: 3011 cctcttcttcttattattctttcttcaaatgcttgtaactcttatggttagagaga 2956



>MDC000748.724 chr17:1443321..1449646
          Length = 6326

 Score =  682 bits (344), Expect = 0.0
 Identities = 353/356 (99%)
 Strand = Plus / Minus


Query: 1    aaccaaactgtagcacacaattcatcactgctatgacagctaagacattccaaatcacac 60
            ||||||||||||||||||||||||||||||||||||||||||||||||||||||||||||
Sbjct: 4944 aaccaaactgtagcacacaattcatcactgctatgacagctaagacattccaaatcacac 4885


Query: 61   aagtttacatacattgggataaaacccacagttattcattgttaaatataattacaataa 120
            ||||||||||||||||||||||||||||||||||||||||||||||||||||||||||||
Sbjct: 4884 aagtttacatacattgggataaaacccacagttattcattgttaaatataattacaataa 4825


Query: 121  gttgattttgttggatgcaaccatccaactactatgatccttcagcgcccaatgttgga 180
            ||||||||||||||||||||||||||||||||||||||||||||||||||||||||||||
Sbjct: 4824 gttgattttgttggatgcaaccatccaactactatgatccttcagcgcccaatgttgga 4765


Query: 181  ttttcaaaggcgaagaaactccatactcaaaatcgtgggcatcggctggagagagattgt 240
            ||||| ||||||||||||||||||||||||||||||||||||||||||||||||||||||
Sbjct: 4764 ttttctaaggcgaagaaactccatactcaaaatcgtgggcatcggctggagagagattgt 4705


Query: 241  cttcgcgtcttgggttgaagtgcgagaccttgaagaatatgagaactggatcatctgggt 300
            ||||||||||||||||||||||||||||||||||||||||||||||||||||||||||||
Sbjct: 4704 cttcgcgtcttgggttgaagtgcgagaccttgaagaatatgagaactggatcatctgggt 4645
```

```
Query: 301  cctcttcttcttattattctttcttcaaaagtttgtaactcttatggttagagaga 356
            |||||||||||||||||||||||||||||| | |||||||||||||||||||||||
Sbjct: 4644 cctcttcttcttattattctttcttcaaatgcttgtaactcttatggttagagaga 4589
```

```
 Score =  111 bits (56), Expect = 8e-23
 Identities = 107/124 (86%), Gaps = 10/124 (8%)
 Strand = Plus / Plus


Query: 198  actccatactcaaaatcgtgggcatcggctggagagagattgtcttcgcg---------t 248
            ||||||||||||||||||||| |||| |||||||||||||| |||| ||         |
Sbjct: 3347 actccatactcaaaatcgtgggaatcgtctggagagagattgccttcccgcgctcagggt 3406


Query: 249  cttgggttgaagtgcgagaccttgaagaatatgagaactggatcatctgggtcctcttct 308
            |||||||||||| || |||| |||||||||||||||||||||||||||||||||||||||
Sbjct: 3407 cttgggttgaagagcaagac-ttgaagaatatgagaactggatcatctgggtcctcttcc 3465


Query: 309  tctt 312
            ||||
Sbjct: 3466 tctt 3469
```

```
>MDC015568.236 chr17:1469063..1475893
          Length = 6831

 Score =  379 bits (191), Expect = e-103
 Identities = 200/203 (98%)
 Strand = Plus / Minus


Query: 1    aaccaaactgtagcacacaattcatcactgctatgacagctaagacattccaaatcacac 60
            |||||||||||||||||||||||||||||| |||||||||||||||||||||||||||||
Sbjct: 1419 aaccaaactgtagcacacaattcatcactggtatgacagctaagacattccaaatcacac 1360


Query: 61   aagtttacatacattgggataaaacccacagttattcattgttaaatataattacaataa 120
            |||||||||||||||||||||||||||||||||||||||||||||||||| ||||||||||
Sbjct: 1359 aagtttacatacattgggataaaacccacagttattcattgttaaatatcattacaataa 1300


Query: 121  gttgattttgttggatgcaaccatccaactactatgatccttcagcgcccaatgttgga 180
            ||||||||||||||||||| ||||||||||||||||||||||||||||||||||||||||
Sbjct: 1299 gttgattttgttggatgctaccatccaactactatgatccttcagcgcccaatgttgga 1240


Query: 181  ttttcaaaggcgaagaaactcca 203
            |||||||||||||||||||||||
Sbjct: 1239 ttttcaaaggcgaagaaactcca 1217
```

```
 Score =  123 bits (62), Expect = 2e-26
 Identities = 103/116 (88%), Gaps = 8/116 (6%)
 Strand = Plus / Plus


Query: 201  ccatactcaaaatcgtgggcatcggctggagagagattgtcttcgcg-------tcttgg 253
            ||||||| ||||||| |||||| |||||||||||||| |||||||||       ||||||
Sbjct: 2478 ccatacttaaaatcgcgggcat-ggctggagagagatagtcttcgcgctcaggatcttgg 2536


Query: 254  gttgaagtgcgagaccttgaagaatatgagaactggatcatctgggtcctcttctt 309
            ||||||||||||||| |||||||||||||||| |||||||||||||||||||||||
Sbjct: 2537 gttgaagtgcgagactttgaagaatatgagaaatggatcatctgggtcctcttctt 2592



>MDC013761.427 chr17:1464328..1479871
         Length = 15544

 Score =  379 bits (191), Expect = e-103
 Identities = 200/203 (98%)
 Strand = Plus / Plus


Query: 1    aaccaaactgtagcacacaattcatcactgctatgacagctaagacattccaaatcacac 60
            |||||||||||||||||||||||||||||| |||||||||||||||||||||||||||||
Sbjct: 9417 aaccaaactgtagcacacaattcatcactggtatgacagctaagacattccaaatcacac 9476


Query: 61   aagtttacatacattgggataaaacccacagttattcattgttaaatataattacaataa 120
            |||||||||||||||||||||||||||||||||||||||||||||||||| ||||||||||
Sbjct: 9477 aagtttacatacattgggataaaacccacagttattcattgttaaatatcattacaataa 9536


Query: 121  gttgattttgttggatgcaaccatccaactactatgatccttcagcgcccaatgttgga 180
            |||||||||||||||||| |||||||||||||||||||||||||||||||||||||||||
Sbjct: 9537 gttgattttgttggatgctaccatccaactactatgatccttcagcgcccaatgttgga 9596


Query: 181  ttttcaaaggcgaagaaactcca 203
            |||||||||||||||||||||||
Sbjct: 9597 ttttcaaaggcgaagaaactcca 9619




 Score =  123 bits (62), Expect = 2e-26
 Identities = 103/116 (88%), Gaps = 8/116 (6%)
 Strand = Plus / Minus


Query: 201  ccatactcaaaatcgtgggcatcggctggagagagattgtcttcgcg-------tcttgg 253
            ||||||| ||||||| |||||| |||||||||||||| |||||||||       ||||||
Sbjct: 8358 ccatacttaaaatcgcgggcat-ggctggagagagatagtcttcgcgctcaggatcttgg 8300
```

```
Query: 254  gttgaagtgcgagaccttgaagaatatgagaactggatcatctgggtcctcttctt 309
            ||||||||||||||| |||||||||||||||| |||||||||||||||||||||||
Sbjct: 8299 gttgaagtgcgagactttgaagaatatgagaaatggatcatctgggtcctcttctt 8244
```
